# Association between cultural capital and health literacy during the COVID-19 pandemic among community residents in China: the mediating effect of social capital

**DOI:** 10.3389/fpubh.2023.1199941

**Published:** 2023-11-01

**Authors:** Yi Luo, Hang Zhao, Huayong Chen, Mimi Xiao

**Affiliations:** School of Public Health, Research Center for Medicine and Social Development, Chongqing Medical University, Chongqing, China

**Keywords:** community residents, cultural capital, social capital, health literacy, mediation effect, COVID-19

## Abstract

**Background:**

Health literacy is crucial for managing pandemics such as COVID-19 and maintaining the health of the population; our goal was to investigate the impact of cultural capital on health literacy during the COVID-19 pandemic among community residents and to further examine the mediating role of social capital in the relationship between cultural capital and health literacy.

**Methods:**

A cross-sectional study was conducted among 1,600 community residents selected in Chongqing, China using a stratified random sampling method. Data were gathered through a questionnaire survey, including sociodemographic characteristics, cultural capital, social capital, and health literacy. Chi-square analysis, one-way ANOVA, *t*-test, and hierarchical linear regression were used to analyze the level of health literacy among community residents and the related elements; the structural equation model (SEM) was used to explore the influential mechanisms of health literacy and explore whether social capital acted as a mediator in the relationship between cultural capital and health literacy.

**Results:**

Cultural capital, community participation, community trust, reciprocity, and cognitive social capital had a significant positive effect on health literacy. In addition, the results of SEM indicated that cultural capital not only directly influences health literacy (*β* = 0.383, 95% CI = 0.265–0.648), but also indirectly influences health literacy through three types of social capital (*β* = 0.175, 95% CI = 0.117–0.465; *β* = 0.191, 95% CI = 0.111–0.406; *β* = 0.028, 95% CI = 0.031–0.174); its mediating effect accounting for 50.7% of the overall effect.

**Conclusions:**

Our results highlight the empirical link between cultural capital and health literacy, and suggest that social capital mediates this connection. These findings suggest that governments and communities should focus on the construction of community cultural capital and provide residents with better social capital to improve their health literacy to prepare for future pandemics.

## 1. Introduction

In December 2019, the first cases of novel coronavirus pneumonia 2019 (COVID-19) were reported (1). Since then, the unexpected coronavirus pneumonia epidemic has spread rapidly around the world as a public health catastrophe and has become a threat in many facets of life with significant negative effects on the economy, society, and politics (2–4). When an epidemic struck, a nation's emergency preparedness and medical standards are put to the test, yet it also serves as a test of the populace's lifestyle and health practices. Health literacy is the key to strengthening the latter (5). More emphasis is placed on residents being the first to take responsibility for their health as the COVID-19 moves into a phase of normalized prevention and management. Therefore, it is necessary to improve residents' health literacy.

The concept of health literacy has been applied by relevant scholars in their research fields. In the field of public health, health literacy is defined as the degree to which individuals have the capacity to obtain, process, and understand basic health information and services needed to make appropriate health decisions, and this degree can make public health decisions that benefit the community (6). The COVID-19 outbreak serves as a litmus test for health literacy, demonstrating that the idea is one of the most crucial individual and societal initiatives of the 21st century for public well-being and health (7). Among the published studies, many scholars have argued that an important part of controlling the COVID-19 pandemic is raising people's health literacy levels (8, 9). Health literacy might encourage and empower people to participate in their health care and adopt practices to protect people from COVID-19 and its negative effects (10). Besides, a cross-sectional study confirmed the importance of health literacy for residents during catastrophic public health crises (5). When compared to those with low health literacy, those with great health literacy practiced more preventative actions (11). China has significant advantages in terms of both its political system and its prevention and control systems when it comes to dealing with large public health catastrophes brought on by emerging infectious illnesses, but the COVID-19 pandemic has highlighted a dearth of national health literacy. For instance, a lot of people wore their masks backward and kept touching them with dirty hands (12). Even though the Internet is very advanced, few individuals pay much attention to health information. These findings demonstrate the underappreciated issue of low health literacy among the general public during the COVID-19 pandemic (13). The COVID-19 pandemic highlights the necessity of improving health literacy and preparing residents for future emergency and non-emergency situations, demonstrating that health literacy can be viewed as a social vaccine. Therefore, conducting a survey on health literacy during the COVID-19 pandemic is of great value, both for managing the COVID-19 now and for preparing residents to guard their health in future pandemics.

Many studies have researched on health literacy, with majority of them conducted in the United States (14, 15). Studies in the past have shown that the factors affecting health literacy are primarily focused on financial hardship, older age, poorer educational level, bad health, heavy use of medical services, low socioeconomic status, and the inability to properly utilize internet information (16–19). These studies mainly focus on the broad concept of health literacy, and fewer studies have investigated health literacy in the context of specific events, such as the COVID-19 pandemic. Thus, despite the growing focus paid to health literacy, it is still valuable to study the factors influencing health literacy from a multidimensional perspective in China, the nation with the largest population.

Three contexts are highlighted in the Institute of Medicine's (IOM) fundamental framework for health literacy—(1) healthcare, (2) education, (3) culture and society—where health literacy can be improved at both the level of the person and the population (11). Most health literacy researches, however, focus on the healthcare and educational levels (12–14), less study has been conducted on health literacy from a cultural standpoint.

A sociological concept, “cultural capital,” was developed by Bourdieu after he summarized Marx's idea of capital. He contends that the allocation of cultural capital among social groupings plays a significant role in the resolution of social issues. After Bourdieu, many scholars critically developed the concept of cultural capital, among which Collins pays attention to the micro level of cultural capital and believes that cultural capital can bring people the micro role of emotional energy under immediate circumstances (20). Collins's framework of cultural capital, which gives greater causal energy and a strong emotional element to cultural capital than Bourdieu version, has been recognized by many scholars and applied to their research (21). So our research is based on Collins' cultural capital framework. Among them, cultural capital can be divided into tangible and intangible. We investigate intangible cultural capital, so “cultural capital” means “the inherited traditions, values, beliefs, etc., which constitute the culture of a group (22).” It exists in the cultural networks and relationships that support human activities, and can promote change when large-scale mobilization is needed. Certain studies showed that cultural capital significantly affects health literacy (23, 24). For instance, Singleton (25) indicated that cultural capital is strongly associated with health literacy. The stronger the cultural capital, the more informed pregnant women are about the health effects of tobacco, according to Afsaneh et al. (26). A society-wide strategy should be used, with a focus on cultural issues, according to a study looking at the development of health literacy in China (27). Although it is crucial to comprehend the role that cultural capital plays in health literacy, there haven't been many studies that address cultural capital in the field of public health in China.

Meanwhile, recent researches have confirmed that social capital and the capacity of social networks, in addition to an individual's abilities, are also associated with health literacy (28). Social capital can not only be a source of knowledge (29), but it can also help people believe in their abilities to find, interpret, and take advantage of health information, which affects health literacy (28). “Social capital” means “the social resources that grow in open social networks or social structures that are based on mutual trust (30).” Social networks, participation, trust, reciprocity, and norms are widely recognized as key elements of social capital (31). In terms of the classification of social capital, the literature usually covers two dimensions: structural and cognitive (32). Since about 2004, it has become much more popular to talk about the three dimensions of structural, cognitive, and relational, and this is now the most generally used and acknowledged framework (33). There is evidence that social capital is vital for health literacy and is highly linked to it (34, 35). For instance, a cross-sectional study conducted in China found a favorable relationship between social capital and health literacy (36). A Ghanaian study showed older persons' oral health literacy was positively impacted by social capital (37). A study done in Korea found that bridging social capital has a substantial moderating impact on the connection between health literacy and self-efficacy regarding health information (28).

The connection between social capital and health literacy has been the subject of a few studies (38, 39), and some scholars suggested that cultural capital may have an impact on social capital (20), but there is no empirical research to clarify the link between cultural capital and health literacy among community residents. Existing studies only emphasize the importance of cultural capital on health literacy, which only briefly discussed the connection between these two factors without examining the mechanisms behind the connected pathways. Besides, no study has investigated the relationship among the three elements simultaneously (i.e., cultural capital, social capital, and health literacy). Therefore, this study aims to explore the effect of cultural capital on health literacy during the COVID-19 pandemic and clarify the mediating role of social capital between cultural capital and health literacy during the COVID-19 pandemic among community residents.

## 2. Material and methods

### 2.1. Study design and participants

**Figure 1:**
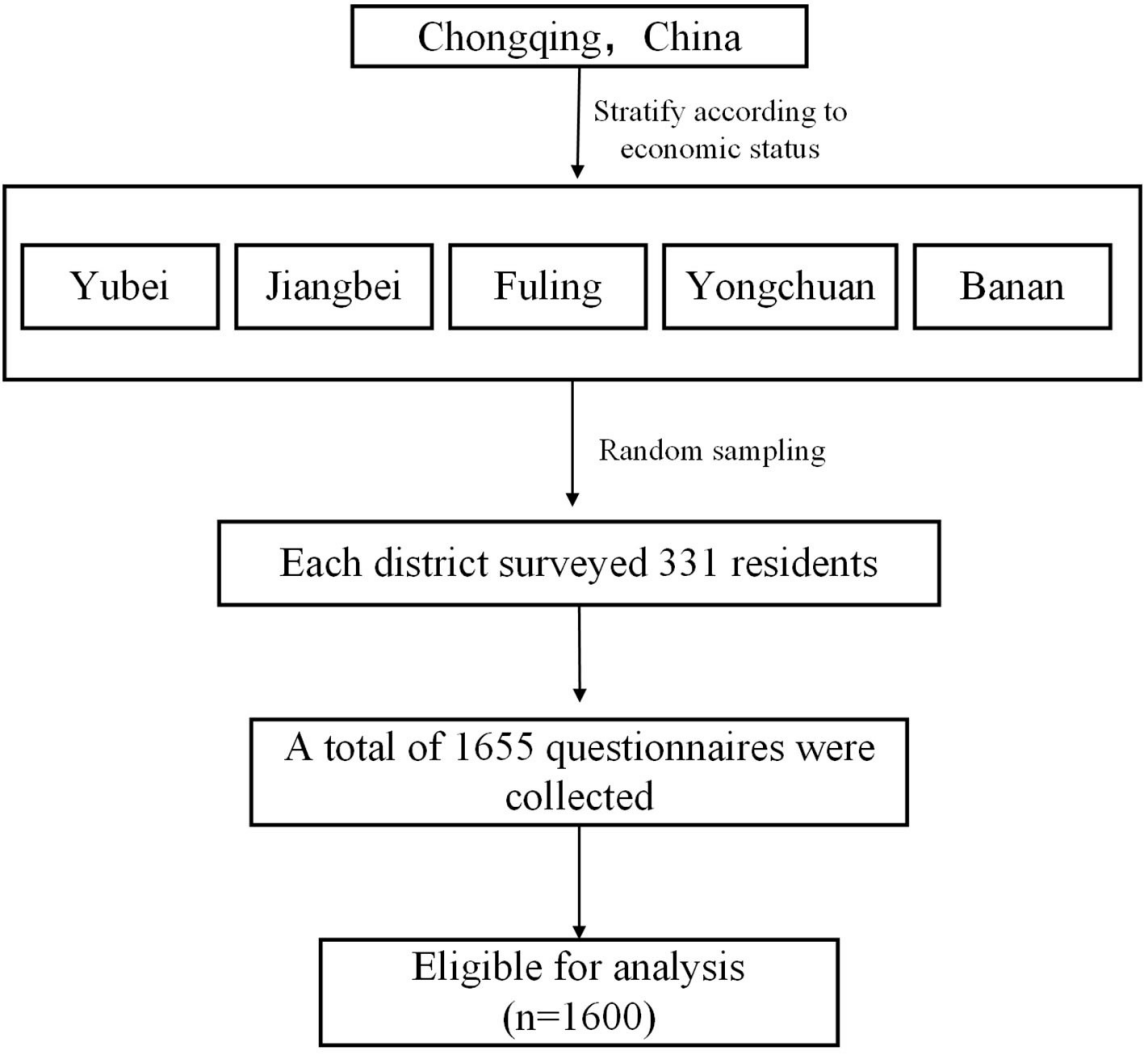
Flow diagram of sample survey selection.

A cross-sectional study was performed among community residents in Chongqing. We used stratified random sampling. First, we stratified according to economic level and used the total GDP of each district as an indicator to measure economic conditions according to the relevant documents published. We selected Yubei and Jiangbei with good economic status, while Fuling and Yongchuan with medium economic status; and Banan with poor economic status. Then, community residents were randomly selected from these districts to conduct the survey. Thus, 355 residents were selected from each district by random sampling. The prerequisites for inclusion were below: (a)being 18–70 years old; (b) being permanent residents; Permanent residents are those who have lived in the community for more than 6 months in the past year; (c) consenting to participate in the survey; (d) having no psychological problems; (e) having the ability to answer questionnaires independently. Finally, we collected 1,600 (96.7%) valid questionnaires (Figure 1).

A preliminary investigation was carried out to determine the precise execution of the research and the final questionnaire design before the full-scale survey. From 1 June to 30 September 2021, the formal inquiry was performed by three fellows and seven university and master's-level students with relevant field research experience. Also, the investigators received training on how to employ the same criteria and techniques; prior to the survey, the participants' informed consent was sought, and any relevant justifications were given during the investigation. We have adopted a series of methods to ensure the reliability of data collection. On the one hand, a one-to-one approach was taken throughout the process of the on-site investigation; on the other hand, the questionnaires were uniformly numbered and double entered after the survey was finished to assure the accuracy of the data entry.

### 2.2. Measured variables

#### 2.2.1. Cultural capital

Based on Collins definition of specific cultural capital, we use three questions to describe cultural capital. The question, “You identify well with the cultural activities in your community,” was used to measure the variable of awareness of identity. The question “Over the past year, you and the residents in your community have shared the same beliefs and goals to fight the pandemic” was used to measure the variable of belief. The question “You follow the core socialist values to mediate interpersonal conflicts and conflicts of interest in your community” was used to measure the variable of values. All items were assessed on a five-point Likert scale (5 = “very much in line with”, 4 = “conform,”, 3 = “don't know”, 2 = “does not match”, and 1 = “very unlikely”). The higher the rating, the greater the cultural capital of community residents. The Cronbach's alpha was 0.956, and confirmatory factor analysis (CFA) reported factor loading was from 0.930 to 0.943, demonstrating high reliability and validity of the scale.

#### 2.2.2. Social capital

The dimensional framework for social capital constructed by Ignacio (40) was used, which describes social capital along three key dimensions: structural, relational, and cognitive. Therefore, a total of three dimensions (structural, cognitive, and relational) and 18 items were included in this study. Specifically, structural social capital included three subdimensions: network size, network interaction, and community participation. There are three items under each subdimension. Relational social capital includes two subdimensions: community trust and reciprocity; there are three items under each subdimension. And cognitive social capital, as a unidimensional variable, contains three items that reflect community norms. All items were assessed on a five-point Likert scale (5 = “very much in line with”, 4 = “conform,”, 3 = “don't know”, 2 = “does not match”, and 1 = “very unlikely”); the higher the rating, the richer the total social capital of community residents. The Cronbach's alpha was 0.979, and confirmatory factor analysis (CFA) reported factor loading was from 0.915 to 0.966, demonstrating high reliability and validity of the scale.

#### 2.2.3. Health literacy

The definition of health literacy involves the ability to acquire, understand and process health information, which corresponds to the theory of knowledge, attitude, and behavior (KAB), so the health literacy evaluation index scale of residents is founded on the theory of KAB. The Communicative and Critical Health Literacy (CCHL) scale developed by Ishikawa et al. (41) was adopted and adapted in conjunction with our research purposes and the reality of COVID-19 pandemic. The scale includes three dimensions: knowledge, attitude, and behavior. For example, the item “You know that WHO defines the novel coronavirus as COVID-19” is in the knowledge dimension; the item “You think the psychological assistance hotline is helpful for psychological counseling during the epidemic” is in the attitude dimension; The item “You will carry out strict hand washing and disinfection every time you go out and come back home” is in the behavioral dimension. There are three items under the knowledge and belief dimensions, respectively, and five items under the behavior dimension, for a total of 11 items on the scale. The scale's items were evaluated using a five-point Likert scale (5 = “very much in line with”, 4 = “conform,”, 3 = “don't know”, 2 = “does not match”, and 1 = “very unlikely”); the higher the rating, the richer the health literacy of community residents. The Cronbach's alpha was 0.978, and confirmatory factor analysis (CFA) reported factor loading was from 0.945 to 0.968, demonstrating high reliability and validity of the scale.

#### 2.2.4. Covariates

The first group of covariates considered were demographic and socioeconomic characteristics, including age, gender (male and female), political status (the masses, communist youth league members, party activists, preparatory party members, communist party members), education (junior high school or below, high school, college, bachelor's degree or above), marital status (unmarried, married, divorced and others), and the type of community they live in (upscale community, general community, older community). The second group consisted of variables of management systems, including risk evaluation management system (have, don't have, don't know). Accountability system for public health emergencies (have, don't have, don't know). The extent to which smart tools work (very helpful, helpful, fair).

### 2.3. Statistical analysis

First, SPSS 26.0 was used for statistical analysis. A two-tailed *P*-value of <0.05 was the threshold for significance. Descriptive statistics like frequency and constituent ratio were used to analyze the data. The t-test and one-way ANOVA were used to analyze the distribution differences of the health literacy score in different sociodemographic characteristics.

Next, to evaluate the relationship among cultural capital, social capital, and health literacy, the correlation coefficients were estimated, and the main variables affecting the health literacy of community residents were examined using hierarchical multiple regression analysis. To account for any potential confounding, all variables that might have an impact on health literacy were included in the model.

Finally, the Structural Equation Model (SEM) was created using AMOS 26.0 to investigate the precise mechanisms underlying the effects of cultural capital, structural social capital, relational social capital, and cognitive social capital on health literacy. We evaluated the fit of the hypothesized model to ensure it fits best with the sample data. The Bootstrap approach was used to estimate both direct and indirect effects in order to calculate confidence ranges for each effect. The corresponding effect is significant if the 95% confidence interval (CI) does not contain 0.

## 3. Result

### 3.1. Characteristics of the sample

Table 1 presents the participant's sociodemographic information. In this study, women accounted for 71.2% of the respondents, and they were mainly in the age group of 31–40 years old (46.3%); in terms of political appearance, the majority were the masses (56.6%), and education was bachelor's degree or above (72.0%); the majority of people were married (80.9%); and nearly 72.9% of the communities where they lived had a risk evaluation management system; 80% of the communities where they lived have a clear accountability system for public health emergencies; most people (77.8%) think smart tools are very helpful in preventing and controlling the coronavirus pneumonia epidemic; and the majority of residents live in the general community (63.0%).

**Table 1:** Descriptive statistics of the participants (*N* = 1,600).

**Variables**	**Category**	***N* (%)**	**Health literacy**	**t/F**	***P***
Gender	Man	461 (28.8)	4.15 ± 0.87	3.329^a^	0.329
	Woman	1,139 (71.2)	4.29 ± 0.73		
Age	18–30	320 (20.0)	4.21 ± 0.77	0.719^b^	0.540
	31–40	741 (46.3)	4.26 ± 0.29		
	41–50	434 (27.1)	4.29 ± 0.77		
	>50	105 (6.6)	4.20 ± 0.80		
Political Appearance	The masses	906 (56.6)	4.26 ± 0.81	3.794^b^	0.004
	League member	231 (14.4)	4.10 ± 0.74		
	Active member of the party	43 (2.7)	4.34 ± 0.54		
	Preparatory party members	40 (2.5)	4.49 ± 0.75		
	Communist party members	380 (23.8)	4.30 ± 0.74		
Education	Junior high school or below	21 (1.3)	3.40 ± 0.90	30.770^b^	< 0.001
	High school	104 (6.5)	3.70 ± 0.93		
	College	323 (20.2)	4.22 ± 0.80		
	Bachelor's degree or above	1,152 (72.0)	4.32 ± 0.72		
Marital status	Unmarried	258 (16.1)	3.81 ± 0.72	58.937^b^	< 0.001
	Married	1,295 (80.9)	4.35 ± 0.75		
	Divorced and others	47 (2.9)	3.96 ± 0.89		
Risk evaluation management system	Have	1,167 (72.9)	4.43 ± 0.68	140.801^b^	< 0.001
	Don't have	139 (8.7)	3.95 ± 0.86		
	Don't know	294 (18.4)	3.68 ± 0.78		
Accountability system for public health emergencies	Have	1,280 (80.0)	4.41 ± 0.70	169.107^b^	< 0.001
	Don't have	90 (5.6)	3.96 ± 0.82		
	Don't know	230 (14.4)	3.50 ± 0.73		
The extent to which smart tools work	Very helpful	1,245 (77.8)	4.44 ± 0.69	214.714^b^	< 0.001
	Helpful	251 (15.7)	3.67 ± 0.67		
	Fair	104 (6.5)	3.39 ± 0.71		
Type of community you live in	Upscale community	158 (9.9)	4.29 ± 0.86	0.274^b^	0.761
	General community	1,008 (63.0)	4.24 ± 0.78		
	Older community	434 (27.1)	4.26 ± 0.74		

a t-test.

b one-way ANOVA test.

Univariate analysis showed residents' health literacy ratings varied significantly (*P* < 0.05) depending on their level of political appearance, education, marital status, risk evaluation management system, accountability system, and the extent to which smart tools work.

### 3.2. Correlation analysis among key variables

Table 2 shows the result of the bivariate correlation, cultural capital has a positive correlation with health literacy (*r* = 0.906, *P* < 0.05), since these two variables are highly correlated, we have included a scatter plot in the Supplementary material. The subdimension of structural social capital was positively correlated with health literacy (*r* = 0.737–0.879, *P* < 0.01), and the correlation degree of community participation was the highest (*r* = 0.879); the subdimension of relational social capital was positively correlated with health literacy (*r* = 0.873–0.886, *P* < 0.01), among which community trust had the highest correlation degree (*r* = 0.886); cognitive social capital also had a positive relationship with health literacy (*r* = 0.869, *P* < 0.01).

**Table 2:** Correlation coefficient of each variable.

**Variables**	**1**	**2**	**3**	**4**	**5**	**6**	**7**	**8**
1. Cultural capital	1							
2. Network interaction	0.735^**^	2						
3. Network size	0.775^**^	0.748^**^	3					
4. Community participation	0.846^**^	0.735^**^	0.753^**^	4				
5. Community trust	0.869^**^	0.744^**^	0.776^**^	0.866^*^	5			
6. Reciprocity	0.869^**^	0.744^**^	0.772^**^	0.850^**^	0.875^**^	6		
7. Cognitive social capital	0.894^**^	0.729^*^	0.784^**^	0.807^**^	0.841^*^	0.870^**^	7	
8. Health literacy	0.906^**^	0.737^*^	0.775^*^	0.879^*^	0.886^*^	0.873^*^	0.869^*^	8

* P < 0.01.

** P < 0.05.

### 3.3. Relationship between cultural capital and health literacy

Health literacy was predicted using hierarchical multiple linear regression. To begin, the unordered categorical data that were statistically significant in the one-way ANOVA of health literacy were represented by dummy variables. After that, five models were used in hierarchical multiple regression analysis using health literacy as the dependant variable: (1) sociodemographic characteristics; (2) sociodemographic characteristics + cultural capital; (3) sociodemographic characteristics + cultural capital + structural social capital; (4) sociodemographic characteristics + cultural capital + structural social capital + relational social capital; (5) sociodemographic characteristics + cultural capital + structural social capital + relational social capital + cognitive social capital. Results showed that ΔR^2^ was statistically significant when sociodemographic characteristics, cultural capital, structural social capital, relational social capital, and cognitive social capital were entered into the regression equation. By comparing the changes in the ΔR^2^, the influence of cultural capital on health literacy was greater than that of sociodemographic factors and social capital, making up 51.3% of the variation in health literacy (Table 3).

**Table 3:** Impacts of cultural capital on health literacy among community residents.

**Variable**	**Model 1**	**Model 2**	**Model 3**	**Model 4**	**Model 5**
	**Standardized** ***B*****eta**	**Standardized** ***B*****eta**	**Standardized** ***B*****eta**	**Standardized** ***B*****eta**	**Standardized** ***B*****eta**
**Political appearance (ref**. = **masses)**					
Communist youth league members	0.047^*^	0.010	0.012	0.005	0.006
Party activists	0.032	0.010	0.014	0.012	0.011
Preparatory party members	0.035^*^	0.009	0.004	0.006	0.006
Communist party members	0.014	0.002	0.005	0.004	0.005
**Education (ref**. = **junior high school or below)**					
Senior high school	0.011^*^	0.033	0.056^*^	0.065^*^	0.058^*^
College	0.246^**^	0.078^*^	0.109^*^	0.108^*^	0.097^*^
Bachelor's degree or above	0.300^**^	0.103^*^	0.134^**^	0.130^**^	0.119^**^
**Marital status (ref**. = **unmarried)**					
Married	0.098^**^	−0.017	0.009	0.010	0.014
Divorced and others	0.021	−0.006	0.008	0.007	0.004
**Risk evaluation management system (ref**. = **None)**					
Have	0.136^**^	0.011	0.012	0.009	0.006
Don't know	−0.019	0.004	0.011	0.011^**^	0.017
**Accountability system for public health emergencies (ref**. = **None)**					
Have	0.053	0.019	0.015	0.018	0.016
Don't know	−0.162^**^	−0.020^**^	−0.012^**^	−0.009^**^	−0.008
The extent to which smart tools work	0.296^**^	0.082^**^	0.041^**^	0.038^**^	0.040^**^
Cultural capital		0.854^**^	0.503^**^	0.381^**^	0.329^**^
**Structural social capital**					
Network interaction			0.037^*^	0.008	0.005
Network size			0.079^**^	0.041^*^	0.028
Community participation			0.336^**^	0.229^**^	0.231^**^
**Relational social capital**					
Community trust				0.191^**^	0.183^**^
Reciprocity				0.117^**^	0.084^**^
**Cognitive social capital**					0.116^**^
*R^2^*	0.313	0.830	0.872	0.883	0.885
*F*	52.935^**^	521.069^**^	608.107^**^	607.006^**^	589.118^**^
Δ*R^2^*	0.319	0.513	0.042	0.011	0.002
Δ*F*	52.395^**^	4,821.187^**^	176.637^**^	76.232^**^	27.514^**^

* P < 0.05.

** P < 0.001.

Specifically, the results of the fifth model's comparison of independent variables showed that health literacy increased with increasing cultural capital score (β = 0.329, *P* < 0.001); in the structural social capital dimension, health literacy score increases in direct proportion to community participation score (β = 0.231, *P* < 0. 001); in the relational social capital dimension, health literacy increased with increasing community trust and reciprocity score (β = 0.183, *P* < 0.001; β = 0.084, *P* < 0.001); the higher the cognitive social capital score, the higher the health literacy score (β = 0.116, *P* < 0. 001).

### 3.4. Model construction

**Figure 2:**
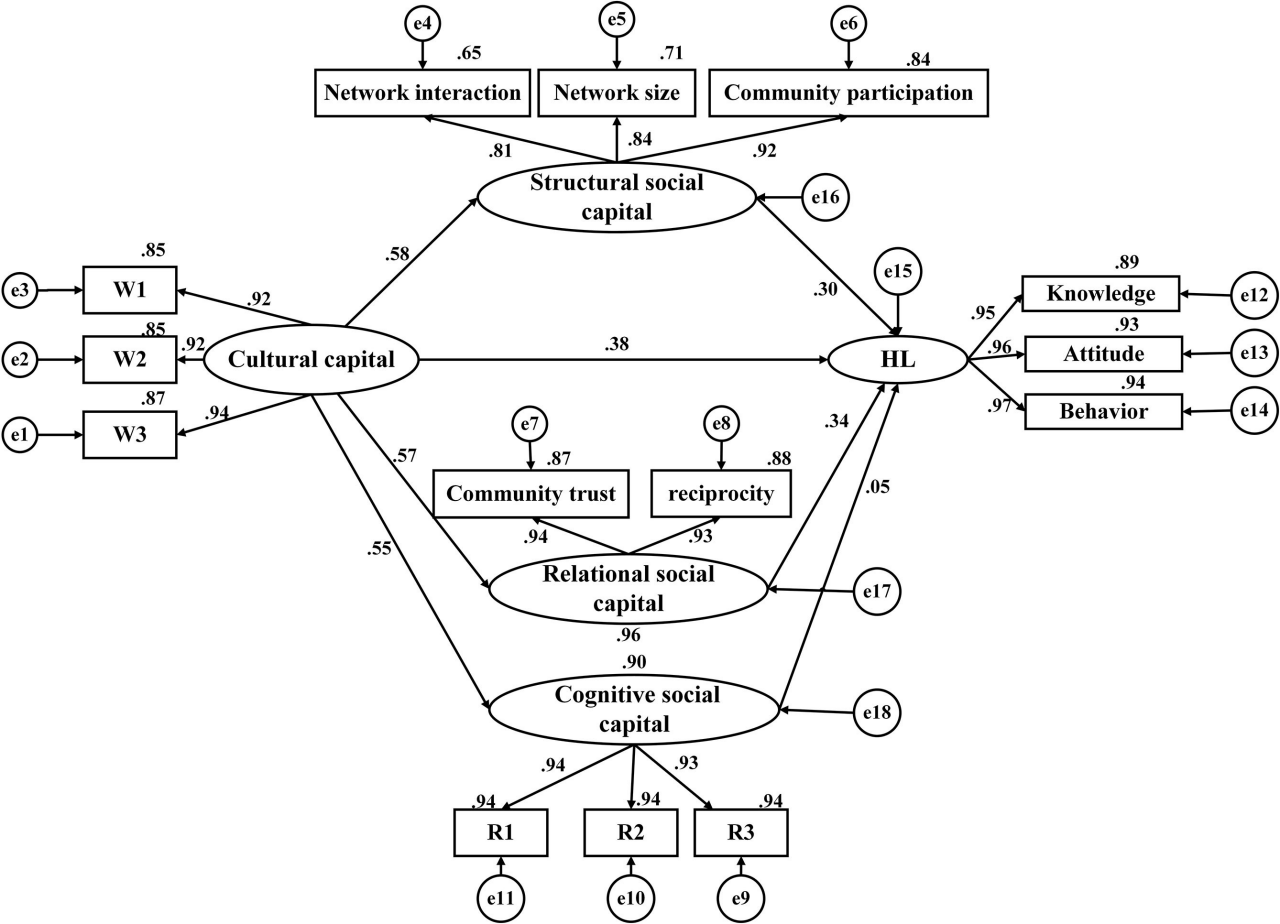
Structural equation model of health literacy.

Structural Equation Model was built in order to further explore the influence mechanism of cultural capital and social capital on health literacy among community residents (Figure 2). Latent variables in the model include cultural capital, three different types of social capital, and health literacy. The three observed variables of cultural capital are three items under the dimension. The three observed variables of structural social capital are network interaction, network size, and community participation; the two observed variables of relational social capital include community trust and reciprocity; the three observed variables of cognitive social capital are three items under the dimension, and the three observed variables of health literacy include knowledge, attitude, and behavior. The model fit results showed that all pathways were statistically significant (*P* < 0.001). The model's estimation procedure uses the maximum likelihood approach. χ2/*df* = 2.281, RMSEA = 0.012, GFI = 0.992, NFI = 0.954, CFI = 0.989, TLI = 0.994, the goodness of fit statistics of the model indicated that the created theoretical models fit the data well (Table 4).

**Table 4:** The fitting results of SEM.

**Fit indices**	**Standards of fit indices**	**Model fit**
*χ^2^*/*df*	1 < *χ^2^*/*df* < 3 good	2.281
RMSEA	< 0.08 acceptable	0.012
GFI	>0.9 acceptable	0.992
NFI	>0.9 acceptable	0.954
CFI	>0.9 acceptable	0.989
TIL	>0.9 acceptable	0.994

### 3.5. Path analysis for health literacy

By standardizing the effects, we discovered that cultural capital and three different types of social capital had positive effects on the health literacy of community residents, with standardized path coefficients being 0.777, 0.303, 0.336, and 0.051 (*P* < 0.001), respectively. Cultural capital contributed positive effects on structural social capital, relational social capital, and cognitive social capital, with the coefficients being 0.578, 0.569, and 0.547 (*P* < 0.001), respectively. Moreover, cultural capital had not only direct effects on health literacy (β = 0.383), but also indirect effects through three types of social capital as the mediators (β = 0.175; β = 0.191; β = 0.028). Besides, three types of social capital had only a direct effect on health literacy (Table 5).

**Table 5:** Path coefficients for health literacy.

**Relations between variables**	**Standardized direct effect**	**Standardized indirect effect**	**Standardized total effect**	**p-value**
Cultural capital → HL	0.383	0.394	0.777	< 0.01
Cultural capital → structural social capital	0.578	/	0.578	< 0.01
Cultural capital → relational social capital	0.569	/	0.569	< 0.01
Cultural capital → cognitive social capital	0.547	/	0.547	< 0.01
Structural social capital → HL	0.303	/	0.303	< 0.01
Relational social capital → HL	0.336	/	0.336	< 0.01
Cognitive social capital → HL	0.051	/	0.051	< 0.01

### 3.6. Mediation effect analysis

The bootstrap analyses showed that the total effects of cultural capital on health literacy were 0.777 (95% CI: 0.046–0.523). The sizes of the direct effect of cultural capital on health literacy were 0.383 (95% CI: 0.045–0.510); the indirect mediation effects via three types of social capital were 0.175 (95% CI: 0.117–0.465), 0.191 (95% CI: 0.111–0.406), 0.028 (95% CI: 0.031–0.174). The 95% CI did not contain 0, hence these effects are significant. Thus, we can conclude that social capital played the mediating role between cultural capital and health literacy, and the mediation effect of social capital makes up 50.7% of the total effect (Table 6).

**Table 6:** Mediating effects of social capital.

**Paths**	**Standardized coefficient**	**Bootstrap 95%CI**		***p***
		**Lower bounds**	**Upper bounds**	
**Total effect**				
Cultural capital → HL	0.777	0.046	0.523	< 0.05
**Direct effect**				
Cultural capital → HL	0.383	0.045	0.510	< 0.05
**Indirect effect**				
Cultural capital → structural social capital → HL	0.175	0.117	0.465	< 0.05
Cultural capital → relational social capital → HL	0.191	0.111	0.406	< 0.05
Cultural capital → cognitive social capital → HL	0.028	0.031	0.174	< 0.05

## 4. Discussion

The purpose of the study is to explore the association between cultural capital and health literacy during the COVID-19 pandemic among community residents in Chongqing, China; and to examine the mediation role of social capital between cultural capital and health literacy. As far as we know, this work is the first to identify the mediation role of social capital between cultural capital and health literacy among community residents. Our findings indicate that cultural capital not only influences health literacy during the COVID-19 pandemic among community residents directly but also indirectly through social capital.

### 4.1. Cultural capital and social capital under the pandemic

Our results show that during the pandemic, the score for cultural capital (4.198 ± 0.810) was higher than Natalie Ross's findings (42) conducted before the pandemic and the score for social capital (3.693 ± 0.683) was higher than Fuyong Hu's findings (43) conducted before the pandemic. These results indicate that the pandemic has promoted the improvement of the cultural capital and social capital of the community residents. The reason may be that, on the one hand, under the impetus of the pandemic, China has actively strengthened the education of values and the cultivation of anti-epidemic spirit. In particular, Chongqing issued policy documents to promote the construction of values, and held various forms of values education activities, resulting in residents having a solid concept of values and a firm sense of belief in fighting the epidemic. On the other hand, in the outbreak of the epidemic, people will lack a sense of security, feel flustered, and under the external stimulation of facing the crisis, people are more likely to produce cohesion (44). Therefore, under such circumstances, social capital has been effectively developed and social resources have been fully mobilized.

### 4.2. Cultural capital and health literacy

This study found that the influence of cultural capital on health literacy during the COVID-19 pandemic is favorable, the health literacy increased with increased cultural capital, consistent with existing studies (45, 46). This result is consistent with the health belief model (HBM), which argues that perception, values, and beliefs are the most significant examples of implicit activity that directly influences people to adopt a particular behavior (47). According to Shuaijun Guo, health literacy is sensitive to a wide range of cultural contexts and may be the result of interactions affected by individual health skills and the social environment (48). Specifically, the item “In the past year, you shared common beliefs and goals with residents in your community to fight against COVID-19 epidemics” showed a more significant impact on health literacy, which is consistent with previous research (49, 50). This occurrence may be related to the psychological toll that the COVID-19 outbreak has had on people (51), people may experience worry and depression and so be less inclined to follow the advised prevention measures (52). When they share a common set of beliefs with the neighbors, it will help them to provide more emotional comfort and alleviate negative emotions for residents, and be more receptive to information about epidemic prevention conveyed by the community (53). This will help them adopt proper epidemic prevention actions and improve their health literacy during the COVID-19 pandemic. Similar research indicates that beliefs, cultural values, and group identity serve as effective information filters (54). Previous studies are limited to the elaboration of the importance of cultural capital on health literacy (55–57), and lack of empirical studies, our study provides empirical support to extend this well.

### 4.3. Social capital and health literacy

We distinguished three types of social capital and examined the effect of each type on health literacy separately. Our results indicate that all types of social capital were positively correlated with health literacy. Previous researches have consistently shown that social capital and health literacy are strongly correlated (58, 59). Specifically, in the structural social capital dimension, community participation has statistically significant associations with health literacy, because being active in the community increases access to sources of crucial health-related information (35), which can increase health promotion options during the COVID-19 pandemic. According to some research, people who are more involved in community activities can gain more health information from interactions with other residents and community health promotion, thus improving their health literacy (60, 61). Communities and neighborhoods with high levels of social cohesion frequently have inbuilt control mechanisms that promote health and keep residents from engaging in behaviors that are harmful to their health (62).

In terms of relational social capital, we discovered that, in line with prior research, health literacy rose with higher community trust and reciprocity (49, 63). The range of resources mobilized when people need to seek out health information can be expanded by relational social capital (64). During the COVID-19 pandemic, having a strong network of relationships among community members facilitated effective communication and information sharing (65), thus enhancing people's health literacy. First, community trust had a favorable impact on health literacy. This effect may be explained by the fact that community residents are less receptive to the health advice they receive from their neighbors and communities when they distrust their social networks, and information utilization is lower. An earlier study in Ghana found that adults often relied on their informal networks when seeking health care, which is frequently explained by a high level of social network trust (37). Besides, research has indicated that residents who practice reciprocity build stronger social networks and have higher levels of self-efficacy while exchanging health information (28). In this way, the exchange of health information can be used to better improve their health literacy (66).

Finally, we found that cognitive social capital also showed a positive effect on health literacy. Some studies have shown that cognitive social capital can control unhealthy behaviors in an informal way to benefit people's health (29). During the COVID-19 pandemic, residents are prone to take some unrecognized and unhealthy behaviors under the catalyst of panic, communities with strict daily rules and regulations can help residents reject false information and provide a good environment for information dissemination; residents are better able to resist rumors and adopt correct behavior in such an environment (67), thus improving their health literacy. For example, during the epidemic, rumors circulate that a specific medication can treat COVID-19, which tends to mislead residents; communities with strict rules and regulations will disseminate illuminating information and provide accurate knowledge about epidemic prevention, which can aid residents in correctly enhancing their health literacy.

### 4.4. The mediating role of social capital

We found that cultural capital promoted health literacy through strengthening social capital. In other words, those with greater cultural capital will also have richer social capital, hence improving their health literacy. This study's findings are explicable by the terror management theory (TMT), which contends that people react favorably to things that uphold their cultural values and that cultural values act as anxiety buffers for people, enabling them to respond positively and having an effect on their cognition and behavior (68). In addition, Ivan light, a great pioneer in sociology, directly pointed out in his article from the perspective of entrepreneurship that it is difficult for social capital to play a role beyond the support of cultural capital. Cultural capital can play a supportive role in social capital, which further emphasizes the symbiotic status of cultural capital and social capital. In a word, cultural capital can promote social capital, and social capital can play the role of bridge and bond, which is consistent with our findings (69). People are prone to dread during the COVID-19 pandemic, but with the correct cultural values at the forefront, cohesion will be strengthened, social capital will be effectively developed, and people will be able to access more useful health information (70), thereby enhancing health literacy. Relational social capital has the strongest mediating effect among the three categories of social capital, which is in line with previous research (64). Cultural elements, such as beliefs, were found to be important predictors of trust capital in a large sample and to have a favorable impact on health in empirical research of southern Africa (71). Conflicts and altercations, for instance, with community workers and other residents during the COVID-19 epidemic are prone to occur when negative emotions act as a catalyst. Residents who uphold the proper cultural values and have a strong sense of community identity can avoid these conflicts and trust each other, have strong interpersonal networks, strengthen their sense of belonging to the community, and develop their social capital systems. As a result, health information and programs are more widely accepted and given more attention, leading to an increase in health literacy. Our results are consistent with Fukuyama's argument (72) that social capital is a melting pot of social resources that is critical to the health of a population and it relies on cultural roots.

### 4.5. Limitations and future studies

The work has a few limitations. First of all, as this was a cross-sectional study, causal conclusions could not be drawn. Cohort follow-up might be used in future studies to acquire longitudinal data, which would provide more conclusive data for causal inference. The second limitation is the representativeness of the sample. Because only Chongqing was conducted for data collection in this study, the results may vary depending on the population or location. Finally, the study's self-reporting and cross-sectional design could have introduced measurement and/or recall bias, which may not reflect the true experiences and perceptions of community residents to a certain extent. In future studies, multiple sources of data can be used to improve the dependability of the questionnaire data, such as paired completion, combinations of self and other ratings, and superior ratings.

## 5. Conclusions

In summary, our findings demonstrate an empirical connection between cultural capital and health literacy among community residents during the COVID-19 pandemic, indicating that cultural capital can promote health literacy during the COVID-19 pandemic; and showing that social capital mediates this connection. Our results provide directions for improving residents' health literacy and suggest that government and community staff should focus on building cultural capital, actively organize cultural events, strengthen the community of values, enhance the sense of internal beliefs, and provide better social capital to improve residents' health literacy to better protect the health of residents in future pandemics.

## Data availability statement

The original contributions presented in the study are included in the article/Supplementary material, further inquiries can be directed to the corresponding author.

## Ethics statement

Written informed consent was obtained from the individual (s) for the publication of any potentially identifiable images or data included in this article.

## Author contributions

YL planned the research, designed the programs, conducted the data collection and analysis, and drafted the manuscript. HZ and HC participated in data collection, assisted data analysis, and gave suggestions for the manuscript writing. MX provided ideas and funding to support this study and helped to revise the manuscript. All authors contributed to the article and approved the submitted version.

## References

[1] Wu F Zhao S Yu B Chen Y Wang W Song Z. A new coronavirus associated with human respiratory disease in China. Nature. (2020) 579:265–9. 10.1038/s41586-020-2008-3 32015508 PMC7094943

[2] Salamzadeh A Dana LP. The coronavirus (COVID-19) pandemic: challenges among Iranian startups. J Small Bus Entrep. (2021) 33:489–512. 10.1080/08276331.2020.1821158

[3] Pereira J Braga V Correia A Salamzadeh A. Unboxing organisational complexity: how does it affect business performance during the COVID-19 pandemic? J Entrep Public Policy. (2021) 10:424–44. 10.1108/JEPP-06-2021-0070

[4] Chirwa GC Dulani B Sithole L Chunga JJ Alfonso W Tengatenga J. Malawi at the crossroads: does the fear of contracting COVID-19 affect the propensity to vote? Eur J Dev Res. (2022) 34:409–31. 10.1057/s41287-020-00353-1 33424140 PMC7781184

[5] Yang P Ou Y Yang H Pei X Li J Wang Y. Research on influencing factors and dimensions of health literacy in different age groups: before and after the COVID-19 era in Chongqing, China. Front Public Health. (2021) 9:690525. 10.3389/fpubh.2021.690525 34552902 PMC8450385

[6] Freedman DA Bess KD Tucker HA Boyd DL Tuchman AM Wallston KA. Public health literacy defined. Am J Prev Med. (2009) 36:446–51. 10.1016/j.amepre.2009.02.001 19362698

[7] Sentell T Vamos S Okan O. Interdisciplinary perspectives on health literacy research around the world: more important than ever in a time of COVID-19. Int J Env Res Pub He. (2020) 17:3010. 10.3390/ijerph17093010 32357457 PMC7246523

[8] Chen C Xu T Chen Y Xu Y Ge L Yao D. Does health literacy promote COVID-19 awareness? Evidence from Zhejiang, China. Front Public Health. (2022) 10:894050. 10.3389/fpubh.2022.894050 36062103 PMC9428714

[9] Spring H. Health literacy and COVID-19. Health Inform Lib J. (2020) 37:171–2. 10.1111/hir.12322 PMC7405264 32672399

[10] Okan O Messer M Levin-Zamir D Paakkari L Sørensen K. Health literacy as a social vaccine in the COVID-19 pandemic. Health Promot Int. (2022). 10.1093/heapro/daab197 35022721 PMC8807235

[11] Rezakhani Moghaddam H Ranjbaran S Babazadeh T. The role of e-health literacy and some cognitive factors in adopting protective behaviors of COVID-19 in Khalkhal residents. Front Public Health. (2022) 10:916362. 10.3389/fpubh.2022.916362 35942262 PMC9356231

[12] Sharma N Bhattacharya S. Role of public health literacy during COVID-19 pandemic, its implications and future recommendations- An analysis from India. J Public Health Prim Care. (2020) 1:13. 10.4103/jphpc.jphpc_8_20

[13] Paakkari L Okan O. COVID-19: health literacy is an underestimated problem. Lancet Public Health. (2020) 5:e249–50. 10.1016/S2468-2667(20)30086-4 32302535 PMC7156243

[14] Rikard RV Thompson MS McKinney J Beauchamp A. Examining health literacy disparities in the United States: a third look at the National Assessment of Adult Literacy (NAAL). BMC Public Health. (2016) 16:975. 10.1186/s12889-016-3621-9 27624540 PMC5022195

[15] Paasche-Orlow MK Parker RM Gazmararian JA Nielsen-Bohlman LT Rudd RR. The prevalence of limited health literacy. J Gen Intern Med. (2005) 20:175–84. 10.1111/j.1525-1497.2005.40245.x 15836552 PMC1490053

[16] Sørensen K Pelikan JM Röthlin F Ganahl K Slonska Z Doyle G. Health literacy in Europe: comparative results of the European health literacy survey (HLS-EU). Eur J Public Health. (2015) 25:1053–8. 10.1093/eurpub/ckv043 25843827 PMC4668324

[17] Protheroe J Whittle R Bartlam B Estacio EV Clark L Kurth J. Health literacy, associated lifestyle and demographic factors in adult population of an English city: a cross-sectional survey. Health Expect. (2017) 20:112–9. 10.1111/hex.12440 26774107 PMC5217902

[18] Amoah PA Musalia J Abrefa Busia K. Health behaviors and health literacy: questing the role of weak social ties among older persons in Rural and Urban Ghana. Front Public Health. (2022) 10:777217. 10.3389/fpubh.2022.777217 35296048 PMC8919952

[19] Lee S-YD Arozullah AM Cho YI. Health literacy, social support, and health: a research agenda. Soc Sci Med. (2004) 58:1309–21. 10.1016/S0277-9536(03)00329-0 14759678

[20] Davies S Rizk J. The three generations of cultural capital research: a narrative review. Rev Educ Res. (2018) 88:331–65. 10.3102/0034654317748423

[21] Collins R. Interaction Ritual Chains. Princeton, NJ: Princeton University Press (2004)

[22] Throsby D. Cultural capital. J Cult Econ. (1999) 23:3–12. 10.1023/A:1007543313370

[23] Shaw SJ Huebner C Armin J Orzech K Vivian J. The role of culture in health literacy and chronic disease screening and management. J Immigr Minor Health. (2009) 11:460–7. 10.1007/s10903-008-9135-5 18379877

[24] Abel T. Cultural capital and social inequality in health. J Epidemiol Commun H. (2008) 62:e13–e13. 10.1136/jech.2007.066159 18572429

[25] Singleton K Krause E. Understanding cultural and linguistic barriers to health literacy. OJIN Online J Issues Nurs. (2009) 14:4. 10.3912/OJIN.Vol14No03Man04 21053716

[26] Tavassoli A Abedi M Modares Gharejedaghi S. Cultural capital and tobacco-related health literacy in pregnant women and the relationship with fetal smoke exposure. Int J High Risk Behav Addict. (2022) 11:e118294. 10.5812/ijhrba.118294

[27] Li YY Lv XF Liang J Dong HJ Chen CG. The development and progress of health literacy in China. Front Public Health. (2022) 10:1034907. 10.3389/fpubh.2022.1034907 36419995 PMC9676454

[28] Kim YC Lim JY Park K. Effects of health literacy and social capital on health information behavior. J Health Commun. (2015) 20:1084–94. 10.1080/10810730.2015.1018636 26166008

[29] Waverijn G Heijmans M Spreeuwenberg P Groenewegen PP. Associations between neighborhood social capital, health literacy, and self-rated health among people with chronic illness. J Health Commun. (2016) 21:36–44. 10.1080/10810730.2016.1179369 27548376

[30] Bjørnskov C. The multiple facets of social capital. Eur J Polit Econ. (2006) 22:22–40. 10.1016/j.ejpoleco.2005.05.006

[31] Bíró É Vincze F Mátyás G Kósa K. Recursive Path Model for Health Literacy: The Effect of Social Support and Geographical Residence. Front Public Health. (2021) 9:724995. 10.3389/fpubh.2021.724995 34650950 PMC8506042

[32] Pettit GS Erath SA Lansford JE Dodge KA Bates JE. Dimensions of social capital and life adjustment in the transition to early adulthood. Int J Behav Dev. (2011) 35:482–9. 10.1177/0165025411422995 22822281 PMC3399414

[33] Villalonga-Olives E Kawachi I. The measurement of social capital. Gac Sanit. (2015) 29:62–4. 10.1016/j.gaceta.2014.09.006 25444390

[34] Hayashi C Maeuma R Yamada K Morioka I. Characteristics of health literacy, social capital, and health behavior acquired through experiences by health promotion volunteers. Nihon Koshu Eisei Zasshi. (2018) 65:107–15. 10.11236/jph.65.3_107 29618708

[35] Blancafort AS Monteserin NR Moral I Roque FM Rojano ILX Coll-Planas L. Promoting social capital, self-management and health literacy in older adults through a group-based intervention delivered in low-income urban areas: results of the randomized trial AEQUALIS. BMC Public Health. (2021) 21:84. 10.1186/s12889-020-10094-9 33413233 PMC7791739

[36] Chen W Zhang C Cui Z Wang J Zhao J Wang J. The impact of social capital on physical activity and nutrition in China: the mediating effect of health literacy. BMC Public Health. (2019) 19:1713. 10.1186/s12889-019-8037-x 31856789 PMC6924071

[37] Amoah PA Koduah AO Gyasi RM Nyamekye KA Phillips DR. Association of health literacy and socioeconomic status with oral health among older adults in ghana: a moderation analysis of social capital. J Appl Gerontol. (2022) 41:671–9. 10.1177/07334648211028391 34225501

[38] Wang JY Gao LF Wang GJ Hu BB. The impact of internet use on old-age support patterns of middle-aged and older adults. Front Public Health. (2023) 10:1059346. 10.3389/fpubh.2022.1059346 36711395 PMC9880032

[39] Liu Y Meng H Tu N Liu D. The relationship between health literacy, social support, depression, and frailty among community-dwelling older patients with hypertension and diabetes in China. Front Public Health. (2020) 8:280. 10.3389/fpubh.2020.00280 32714893 PMC7344226

[40] Castro I Roldán JL. A mediation model between dimensions of social capital. Int Bus Rev. (2013) 22:1034–50. 10.1016/j.ibusrev.2013.02.004

[41] Ishikawa H Nomura K Sato M Yano E. Developing a measure of communicative and critical health literacy: a pilot study of Japanese office workers. Health Promot Int. (2008) 23:269–74. 10.1093/heapro/dan017 18515303

[42] Ross Adkins N Corus C. Health literacy for improved health outcomes: effective capital in the marketplace. J Consum Affairs. (2009) 43:199–222. 10.1111/j.1745-6606.2009.01137.x

[43] Hu F Niu L Chen R Ma Y Qin X Hu Z. The association between social capital and quality of life among type 2 diabetes patients in Anhui province, China: a cross-sectional study. BMC Public Health. (2015) 15:786. 10.1186/s12889-015-2138-y 26276271 PMC4542125

[44] Dauderstadt M Keltek C. Crisis austerity, and cohesion: Europe's stagnating inequalit y. Int J Health Serv. (2015) 45:25–31. 10.2190/HS.45.1.c 26460445

[45] Kale MS Federman AD Krauskopf K Wolf M O'Conor R Martynenko M. The association of health literacy with illness and medication beliefs among patients with chronic obstructive pulmonary disease. PLoS ONE. (2015) 10:e0123937. 10.1371/journal.pone.0123937 25915420 PMC4411058

[46] Peterson NB Dwyer KA Mulvaney SA Dietrich MS Rothman RL. The influence of, health literacy on colorectal cancer screening knowledge, beliefs and behavior. J Natl Med Assoc. (2007) 99:1105–12. 17987913 PMC2574401

[47] Ghorbani-Dehbalaei M Loripoor M Nasirzadeh M. The role of health beliefs and health literacy in women's health promoting behaviours based on the health belief model: a descriptive study. BMC Womens Health. (2021) 21:421. 10.1186/s12905-021-01564-2 34922505 PMC8684276

[48] Guo SJ Yu XM Davis E Armstrong R Riggs E Naccarella L. Adolescent health literacy in Beijing and Melbourne: a cross-cultural comparison. Int J Env Res Pub He. (2020) 17:1242. 10.3390/ijerph17041242 32075168 PMC7068382

[49] Niu Z Qin Z Hu P Wang T. Health beliefs, trust in media sources, health literacy, and preventive behaviors among high-risk Chinese for COVID-19. Health Commun. (2022) 37:1004–12. 10.1080/10410236.2021.1880684 33557620

[50] Cameron LD Lawler S Robbins-Hill A Toor I Brown PM. Political views, health literacy, and COVID-19 beliefs and behaviors: a moderated mediation model. Soc Sci Med. (2023) 320:115672. 10.1016/j.socscimed.2023.115672 36764089 PMC9884608

[51] Duplaga M Grysztar M. The association between future anxiety, health literacy and the perception of the COVID-19 pandemic: a cross-sectional study. Healthcare-Basel. (2021) 9:43. 10.3390/healthcare9010043 33466487 PMC7824811

[52] Briggs AM Jordan JE Buchbinder R Burnett AF O'Sullivan PB Chua J. Health literacy and beliefs among a community cohort with and without chronic low back pain. Pain. (2010) 150:275–83. 10.1016/j.pain.2010.04.031 20603025

[53] Lin Y Hu Z Alias H Wong LP. Knowledge, attitudes, impact, and anxiety regarding COVID-19 infection among the public in China. Front Public Health. (2020) 8:236. 10.3389/fpubh.2020.00236 32574305 PMC7266871

[54] Thomas SB Fine MJ Ibrahim SA. Health disparities: the importance of culture and health communication. Am J Public Health. (2004) 94:2050. 10.2105/AJPH.94.12.2050 15612166 PMC1448585

[55] Rosenbaum AJ Uhl RL Rankin EA Mulligan MT. Social and cultural barriers: understanding musculoskeletal health literacy: AOA critical issues. J Bone Joint Surg Am. (2016) 98:607–15. 10.2106/JBJS.O.00718 27053590

[56] Barros A Santos H Moreira L Santos-Silva F. Translation and cross-cultural adaptation of the cancer health literacy test for Portuguese cancer patients: a pre-test. Int J Env Res Pub He. (2022) 19:6237. 10.3390/ijerph19106237 35627773 PMC9141979

[57] Ingram RR. Using Campinha-Bacote's process of cultural competence model to examine the relationship between health literacy and cultural competence. J Adv Nurs. (2012) 68:695–704. 10.1111/j.1365-2648.2011.05822.x 21895740

[58] Taherian Z Motamedi N. Effect of a 12-week community-based intervention to improve social capital, quality of life, self-care, and health literacy among older people: a quasi-experimental trial. Adv Biomed Res-India. (2022) 11:23. 10.4103/abr.abr_101_21 35720209 PMC9201224

[59] Sentell T Pitt R Buchthal OV. Health literacy in a social context: review of quantitative evidence. Health Lit Res Pract. (2017) 1:e41–70. 10.3928/24748307-20170427-01 31294251 PMC6607851

[60] Nawabi F Krebs F Lorenz L Shukri A Alayli A Stock S. Health literacy among pregnant women in a lifestyle intervention trial. Int J Env Res Pub He. (2022) 19:5808. 10.3390/ijerph19105808 35627343 PMC9141630

[61] Platter H Kaplow K Baur C. The value of community health literacy assessments: health literacy in Maryland. Public Health Rep. (2022) 137:471–8. 10.1177/00333549211002767 33706612 PMC9109546

[62] Miller HN Thornton CP Rodney T Thorpe RJ Allen J. Social cohesion in health: a concept analysis. Adv Nurs Sci. (2020) 43:375–90. 10.1097/ANS.0000000000000327 32956090 PMC8344069

[63] Chen X Hay JL Waters EA Kiviniemi MT Biddle C Schofield E. Health literacy and use and trust in health information. J Health Commun. (2018) 23:724–34. 10.1080/10810730.2018.1511658 30160641 PMC6295319

[64] Abdullah MI Dechun H Ali M Usman M. Ethical leadership and knowledge hiding: a moderated mediation model of relational social capital, and instrumental thinking. Front Psychol. (2019) 10:2403. 10.3389/fpsyg.2019.02403 31708841 PMC6823209

[65] Nooraie RY Warren K Juckett LA Cao QA Bunger AC Patak-Pietrafesa MA. Individual- and group-level network-building interventions to address social isolation and loneliness: a scoping review with implications for COVID 19. PLoS ONE. (2021) 16:e0253734. 10.1371/journal.pone.0253734 34170980 PMC8232435

[66] Chang CW Huang HC Chiang CY Hsu CP Chang CC. Social capital and knowledge sharing: effects on patient safety. J Adv Nurs. (2012) 68:1793–803. 10.1111/j.1365-2648.2011.05871.x 22077142

[67] Freiling I Krause NM Scheufele DA Brossard D. Believing and sharing misinformation, fact-checks, and accurate information on social media: the role of anxiety during COVID-19. New Media Soc. (2023) 25:141–62. 10.1177/14614448211011451 36620434 PMC9805917

[68] Chew P. Big data analysis of terror management theory's predictions in the COVID-19 pandemic. Omega-J Death Dying. (2022) 2022:37592345. 10.1177/00302228221092583 35440220 PMC9024090

[69] Light I Dana L. Boundaries of social capital in entrepreneurship. Entrep Theory Pract. (2013) 37:603–24. 10.1111/etap.12016

[70] Lu Q Chang A Yu G Yang Y Schulz PJ. Social capital and health information seeking in China. BMC Public Health. (2022) 22:1525. 10.1186/s12889-022-13895-2 35948901 PMC9364581

[71] Gershman B. Witchcraft beliefs and the erosion of social capital: evidence from sub-saharan Africa and beyond. J Dev Econ. (2016) 120:182–208. 10.1016/j.jdeveco.2015.11.005

[72] Fukuyama F. Social capital, civil society and development. Third World Q. (2001) 22:7–20. 10.1080/713701144

