# Supplementary figures and images for: Association between cultural capital and health literacy during the COVID-19 pandemic among community residents in China: the mediating effect of social capital
Source: Front Public Health. 2023 Nov 1;11:1199941. doi: 10.3389/fpubh.2023.1199941 (PMC10647931; doi:10.3389/fpubh.2023.1199941)

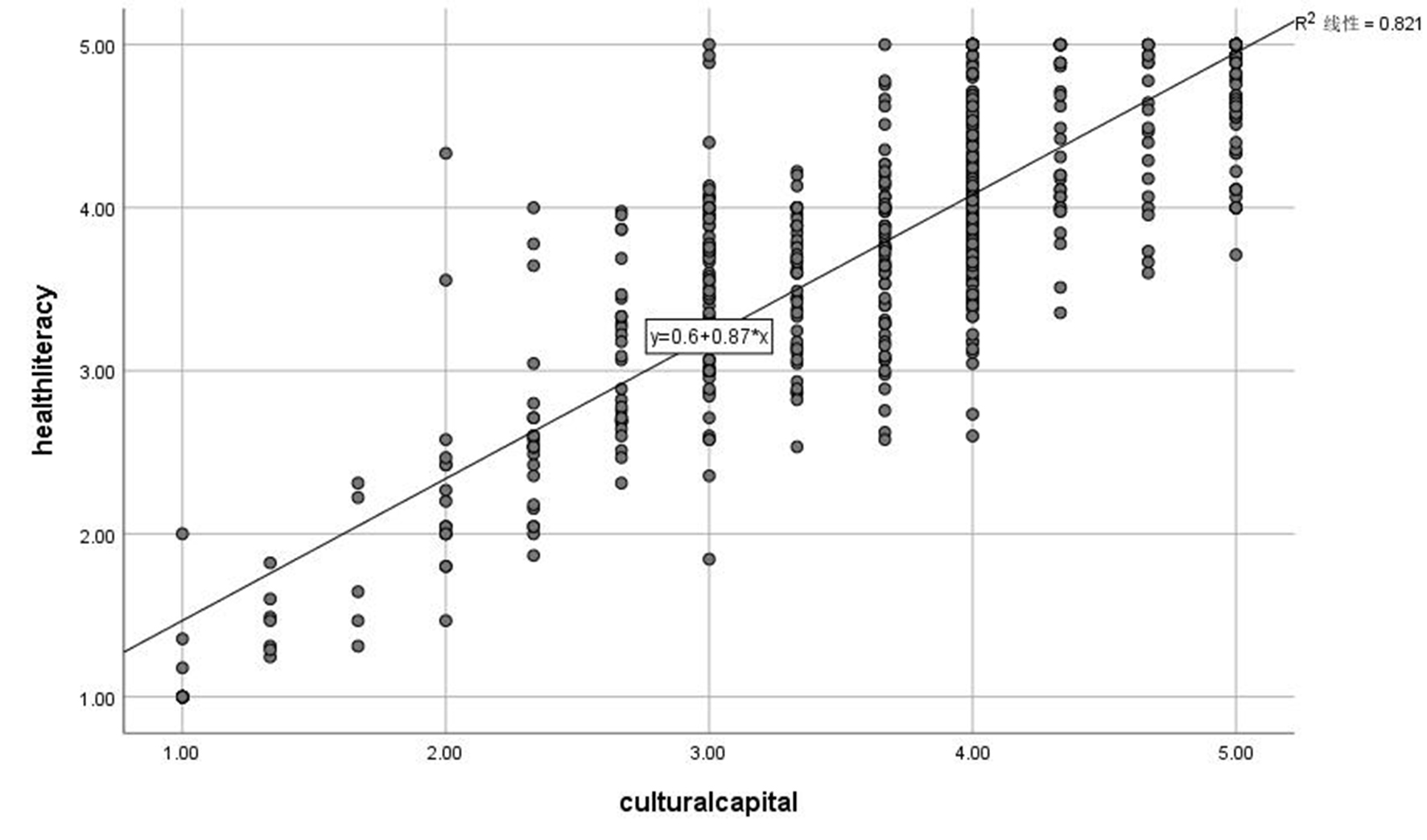

Supplement: Supplementary file 1 [file Image_1.JPEG]
